# Risk factors of deep vein thrombosis in children with osteomyelitis

**DOI:** 10.1080/07853890.2023.2249011

**Published:** 2023-08-25

**Authors:** Yunjian He, Shaoshan Liu, Yuxi Su

**Affiliations:** a Orthopedics Department, Children’s Hospital of Chongqing Medical University, Chongqing, China; b Ministry of Education Key Laboratory of Child Development and Disorders, Chongqing Key Laboratory of Pediatrics, Chongqing, China; c National Clinical Research Center for Child Health and Disorders, Chongqing, China; d China International Science and Technology Cooperation Base of Child Development and Critical Disorders, Jiangxi Hospital Affiliated Children’s Hospital of Chongqing Medical University, Chongqing, China

**Keywords:** Thrombotic, osteomyelitis, methicillin-resistant *Staphylococcus aureus*, children

## Abstract

**Objective:**

To investigate the risk factors for deep vein thrombosis (DVT) in children with osteomyelitis and provide diagnostic and treatment strategies for the prevention, early detection and treatment of DVT.

**Study design:**

The clinical data of nine children diagnosed with osteomyelitis and DVT between July 2012 and March 2021 were collected at our hospital, including age, sex, clinical manifestations, body temperature, coagulation function and other data, as well as the clinical data of 27 children diagnosed with osteomyelitis without DVT during the same period. Thirty-six children were divided into thrombus and thrombus-free groups. The clinical characteristics and risk factors for DVT in children with osteomyelitis were analysed.

**Results:**

Among the 36 children in this study, nine cases of thrombus formation mainly occurred in the femoral vein, popliteal vein and iliac vein, all near the infection site. The main clinical manifestations were lower extremity pain, swelling and pulmonary embolism in three cases. Among them, intensive care unit (ICU) admission, sepsis, higher D-dimer, higher body temperature during hospitalization, and pathogen culture showed that methicillin-resistant *Staphylococcus aureus* (MRSA) was associated with DVT. MRSA was the independent risk factor for DVT.

**Conclusions:**

Admission to ICU, sepsis, higher D-dimer, higher body temperature during hospitalization, and MRSA are risk factors for thrombosis. MRSA is the independent risk factor for DVT. For patients with related risk factors, timely ultrasound examination of the infected site should be considered to achieve early detection and treatment.

## Introduction

The occurrence of thrombosis in the paediatric population is progressively rising. Among these, deep vein thrombosis (DVT) is found to be the most prevalent, presenting an incidence rate between 0.07 and 0.49 per 10,000 [1–3]. DVT, characterized by the accumulation of blood in the venous lumen leading to obstructed blood flow, can become life-threatening if the thrombus dislodges and migrates to the lungs, a condition known as pulmonary embolism (PE). PE and DVT are jointly termed as venous thromboembolisms (VTEs). Physiologically, children typically exhibit a relatively reduced coagulation activity, resulting in a lower incidence of VTE when compared to that in adults. International studies report that the overall occurrence of VTE in children is roughly estimated to be between 0.07 and 0.14 per 10,000. Nonetheless, in the case of hospitalized children, this incidence can escalate by a factor of 100–1000, reaching up to 58 per 10,000 [4]. In addition, because most VTEs are asymptomatic thrombi, which can easily lead to missed diagnoses and misdiagnoses, the actual incidence may be higher [5]. DVT is closely associated with deep vein catheterization, infection, trauma and other factors, with deep vein catheterization being the most common [6]. At present, there are few studies on DVT in children, which have focused on the influence of catheterization on thrombosis. Additionally, there are few related studies on thrombosis caused by infection, and there is a lack of significant data to support the prevention and treatment of thrombosis. Through a retrospective analysis of the clinical data from nine paediatric patients, diagnosed with osteomyelitis and DVT in our institution between July 2012 and March 2021, we aimed to identify thrombosis-related risk factors. The findings of this study will aid in the prevention, early detection and treatment of DVT in children afflicted with osteomyelitis.

## Methods

### General information

Data on children with DVT, who were diagnosed with colour ultrasound, were collected from those admitted to our hospital between July 2012 and March 2021. Children diagnosed with osteomyelitis were screened, their medical records were queried to obtain clinical data, and they were selected as the thrombus group. Children with osteomyelitis who were hospitalized simultaneously without thrombosis were selected as the thrombus-free group at a ratio of one:three. The inclusion criteria for the thrombus group were as follows: ① thrombosis confirmed by ultrasound, with clinical manifestations and test results consistent with osteomyelitis; and ② age from zero to eighteen years. The exclusion criteria for the thrombus group were as follows: ① thrombus related to catheterization and ② incomplete clinical data. The inclusion criteria for the thrombus-free group were: ① hospitalized children diagnosed with osteomyelitis during the same period, whereas the exclusion criterion was incomplete clinical data. Finally, nine and 27 children with and without thrombi, respectively, were included in this study. General data (age, sex), clinical symptoms, thrombus site, coagulation function and other clinical data of the two groups of children were compared and analysed to determine the clinical characteristics and risk factors for thrombus formation in children with osteomyelitis. This study was approved by the Ethics Committee (2022 LUN Review [Research] No. 175), and all participants’ guardians provided informed consent. The study was checked by strobe checklist [7].

### Statistical analysis

SPSS26.0 (IBM, Armonk, NY) was used for statistical analysis. As the data were not normally distributed, measurement data were expressed as median and interquartile range (Q1–Q3), and the Mann–Whitney *U*-test was used for comparison. Categorical data were presented as frequencies, and the Chi-squared test or Fisher’s exact test was used for comparison. Binary logistic regression analysis was performed for the statistically significant factors in the univariate analysis, presenting odds ratios (ORs) and their 95% confidence intervals (CIs). A *p* value of less than .05 was considered statistically significant.

### Patients and methods

Blood counts, C-reactive protein (CRP) measurements and sedimentation measurements were performed on admission; Doppler ultrasonography was performed in children with localized, clinically significant osteomyelitis. Bacteriology included blood cultures on admission and sampling of the infected site for bacterial culture at the time of surgery.

## Results

### Analysis of clinical data

#### Gender and age distribution characteristics of children with thrombosis

Among the nine children with thrombosis, seven were males, accounting for 77.8% (seven out of nine), and two were females, accounting for 22.2% (two out of nine), with a male-to-female ratio of 3.5:1. The age range was 27–160 months, with an average age of 96.6 months and a median age of 125.0 months. The largest proportion of children were in the 10–13 age group, with five out of the nine children falling into this category, representing 55.6% of the total.

#### Distribution characteristics of thrombus sites in children with thrombus

The main sites of thrombosis in the nine children were as follows: femoral vein in eight out of nine cases (88.9%), popliteal vein in six out of nine cases (66.7%), iliac vein in four out of nine cases (44.4%), posterior tibial vein in two out of nine cases (22.2%) and anterior tibial vein in one out of nine cases (11.1%). The locations of the thrombi corresponded to the infection site, and there were seven cases of multi-site thrombosis (Table 1).

**Table 1.** Patients’ demographic characteristics.

	DVT^a^	NO DVT	*p* Value^#^
Age			
Mean age (months)	96.51 ± 18.98	85.51 ± 11.53	.39
Gender			
Male	7	18	.69
Female	2	9	
Thrombus sites			
Femoral vein	8		
Popliteal vein	6		
Iliac vein	4		
Posterior tibial vein	2		
Anterior tibial vein	1		
Clinical features			
Pain	10		
Swelling	8		
Chest pain and dyspnoea	3		

^a^ DVT: deep venous thrombosis.

^#^ *p* < .05 was statistically significant.

#### Clinical features of children with thrombosis

Among the nine children with thrombus, there were clinical symptoms at the site of thrombus, which were caused by the coincidence of the location of the primary disease, osteomyelitis and thrombus. The main clinical manifestations were lower limb swelling, pain and joint pain. At the same time, PE (chest pain and dyspnoea) occurred in three cases (Table 1).

### Analysis of risk factors for thrombosis

Nine children with thrombus were included in the experimental group. During the same period, 27 children without thrombus were included as the control group. Information on clinical data including sex, age, operation, intensive care unit (ICU) admission, body temperature, white blood cell (WBC), CRP, erythrocyte sedimentation rate (ESR) and D-dimer level were collected in both groups. The culture strain analysed was methicillin-resistant *Staphylococcus aureus* (MRSA), with sepsis and maximum body temperature. Univariate analysis showed that children with no surgical treatment, higher body temperature, higher D-dimer level, ICU admission and MRSA were more likely to develop thrombosis than those without thrombosis (Table 2). Factors with a *p* value less than .1 were included in the binary logistics regression analysis, and it was found that MRSA was the only risk factor for thrombosis (Table 3).

**Table 2.** Univariate analysis of risk factors for thrombosis in children with osteomyelitis.

Risk factors	Number	DVT^a^ (*n* = 9)	No DVT^a^ (*n* = 27)	*p* Value^#^
Male	25	7	18	.69
WBC	–	6.10 (3.52–10.61)	6.92 (5.33–9.84)	.41
CRP	–	18 (5.5–54.5)	4 (3.0–31.0)	.41
Median age (months)	–	125 (29.5–150.0)	109 (25.5–140.0)	.39
ESR	–	54 (34.5–100.0)	34 (22–64)	.19
Surgery	23	3	20	.046
ICU staying	4	3	1	.041
Sepsis	17	7	10	.055
D-dimer	–	2.34 (1.32–8.09)	1.07 (0.6–2.3)	.027
Temperature	–	39.8 (38.75–40)	37 (36.9–39.5)	.019
MRSA	7	6	1	.000

^a^ DVT: deep venous thrombosis.

^#^ *p* < .05 was statistically significant.

**Table 3.** Logistic regression analysis of risk factors for thrombosis in children with osteomyelitis.

Risk factors	*B*	S.E.	Wald	*p* ^#^	Exp(*B*)	95%CI
MRSA	6.253	3.042	4.225	.04	519.4	1.337–201736.767
ICU	0.735	2.465	0.089	.766	2.084	0.017–261.201
Surgery	−0.137	1.796	0.006	.939	0.872	0.026–29.426
D-dimer	−0.464	0.337	1.888	.169	0.629	0.325–1.219
Sepsis	−4.172	3.403	1.503	.22	0.015	0–12.158
Temperature	−1.319	0.767	2.959	.085	0.267	0.059–1.202

CI: confidence interval; MRSA: methicillin-resistant *Staphylococcus aureus*.

^#^ *p* < .05 was statistically significant.

## Discussion

The epidemiology, anatomical location and clinical presentation of DVT differ between children and adults. The incidence of DVT in children is much lower than that in adults, and in most cases occur in hospitalized children as serious complications of other diseases and their treatment [8–11]. Reportedly, the incidence of DVT in hospitalized children has increased over the past few decades [12] not only because of great improvements in life support and medical care for critically ill children, but also due to development of more secondary complications, such as thrombosis. In addition, owing to a more comprehensive understanding of thrombi, improvements in the technical skill of clinicians, and improvements in imaging methods, it is easier to detect thrombus formation in children.

Osteomyelitis is reported to affect between 1 and 25 children per 100,000 children each year, with an incidence peak in early childhood, that is more common in boys than in girls. Acute blood-borne osteomyelitis is more common in children than in adults, and severe cases require treatment [13–18]. Currently, *Staphylococcus aureus* infection is the leading cause of osteomyelitis in most paediatric hospitals. MRSA is an extremely common pathogen in hospitals and communities. Every year, paediatric hospitals experience a steady increase in the rate of methicillin-resistant staphylococcal infections [19]. In addition, MRSA causes localized soft-tissue destruction, faster transmission and higher mortality. Patients with MRSA have longer hospital stays and more frequent complications such as DVT and septic PE. In addition, patients with MRSA also have a higher incidence of abscesses and subperiosteal infections requiring surgical intervention. Antibiotics are initiated early in the course of the disease. Even when sepsis is well-diagnosed, blood cultures should be performed before prophylactic antibiotics are administered, to better control the infection.

DVT is relatively rare in children. In children diagnosed with osteomyelitis, lower extremity swelling and pain can be observed during physical examination, and osteomyelitis combined with DVT can be confirmed by performing lower extremity colour ultrasound. The age groups with a high incidence of DVT in children are infants (20%) and adolescents (50%), which was consistent with the age characteristics of the nine cases in our study. Although the total incidence was low, it showed an increasing trend annually [18]. The mechanism by which infections lead to DVT has not yet been fully elucidated. Currently, it is generally believed that the mechanism is as follows: infection causes the host to produce a strong defence response to invading pathogens, triggers coagulation function activation through endothelial cell injury and increase in inflammatory markers, and inhibits fibrinolysis, thus leading to platelet aggregation and thrombosis [20]. The symptoms of DVT and osteomyelitis in children were similar in our study. We found that when osteomyelitis was combined with DVT, all thrombi were located near the primary site, making diagnosis difficult. DVT can aggravate the primary disease of osteomyelitis in children and can easily lead to PE after thrombus detachment, thereby increasing the risk of death. In our nine cases of children with thrombus, three developed PE, which may lead to death at any time; therefore, early diagnosis is crucial. Studies analysing the relationship between childhood osteomyelitis and DVT have shown that children with osteomyelitis who develop DVT, are more likely to have elevated inflammatory markers and a more complex hospitalization course, including longer hospital stays, more surgical interventions, and more frequent ICU visits [21,22]. We also found similar results; in children with osteomyelitis, the thrombus group had a longer hospital stay. The proportion of children aged 30 days in the group with thrombosis was significantly higher than in the thrombus-free group, with six out of nine cases (approximately 66.7%) versus five out of 27 cases (approximately 18.5%). The children in the thrombosis group were also more likely to be admitted to the ICU, with three out of nine cases (approximately 33.3%), compared to only one out of 27 cases (approximately 3.7%) in the thrombus-free group. We did not observe the same results with additional surgical interventions. Reportedly, DVT is more common in children with osteomyelitis when the pathogenic organism is MRSA [23], which agrees with our results. In children with osteomyelitis, the incidence of the pathogenic microorganism MRSA was significantly higher in the thrombus group, with six out of nine cases (approximately 66.7%) compared to only one out of 27 cases (approximately 3.7%) in the thrombus-free group; thus indicating that if MRSA is detected through bacterial culture, alertness to the occurrence of DVT is necessary, and select vascular ultrasound for diagnostic examination should be performed at the earliest possible.

In this study, there was little difference in the mean age between patients with or without DVT (96.56 month years in the thrombus group vs. 85.51 month in the thrombus-free group, respectively). Hollmig et al. [23] reported that the average age of patients with osteomyelitis with DVT was 10.9 years, which was significantly higher than that of patients without DVT. This is inconsistent with our findings.

In this study, the sex of children with DVT was mostly male (male:female, seven:two), which was not significantly different from that of children without thrombosis (male:female, two:one). This predominance of males has also been reported in literature; all patients reported by Gorenstein et al. [24] and eight of the eleven patients reported by Hollmig et al. [23] were boys.

The primary limitation of this study is its retrospective design; children with osteomyelitis were not monitored or followed up. Another limitation is the lack of prospective clinical or radiological studies to detect DVT, as most are incidental findings on infection-related imaging. Few patients present with the clinical symptoms of DVT, except for pain and swelling of the limbs caused by osteomyelitis [20]. Therefore, the actual prevalence of DVT in children with OM may have been underestimated or overestimated. Prospective studies are required to determine the incidence of DVT in children with osteomyelitis.

## Consent form

Informed consent for publication of photographs was obtained from all study participants. The Ethics Committee of the Children’s Hospital of Chongqing Medical University approved the study.

## Data Availability

All data generated or analysed during this study are included in this article. Further enquiries can be directed to the corresponding author.
